# Association between manganese superoxide dismutase promoter gene polymorphism and breast cancer survival

**DOI:** 10.1186/bcr1532

**Published:** 2006-07-19

**Authors:** Robert CG Martin, Jiyoung Ahn, Susan A Nowell, David W Hein, Mark A Doll, Benjamin D Martini, Christine B Ambrosone

**Affiliations:** 1 Division of Surgical Oncology, Department of Surgery, University of Louisville School of Medicine, Louisville, Kentucky, USA; 2 Nutritional Epidemiology Branch, Division of Cancer Epidemiology and Genetics, National Cancer Institute, NIH, DHHS, Bethesda, Maryland, USA; 3 Department of Epidemiology, Roswell Park Cancer Institute, Buffalo, New York, USA; 4 Division of Molecular Epidemiology, National Center for Toxicological Research, Jefferson, Arkansas, USA; 5 Department of Pharmacology and Toxicology, and the James Graham Brown Cancer Center, University of Louisville School of Medicine, Louisville, Kentucky, USA

## Abstract

**Background:**

Manganese superoxide dismutase (MnSOD) plays a critical role in the detoxification of mitochondrial reactive oxygen species, constituting a major cellular defense mechanism against agents that induce oxidative stress. A genetic polymorphism in the mitochondrial targeting sequence of this gene has been associated with increased cancer risk and survival in breast cancer. This base pair transition (-9 T > C) leads to a valine to alanine amino acid change in the mitochondrial targeting sequence. A polymorphism has also been identified in the proximal region of the promoter (-102 C>T) that alters the recognition sequence of the AP-2 transcription factor, leading to a reduction in transcriptional activity. The aim of our study was to investigate possible associations of the -102 C>T polymorphism with overall and relapse-free breast cancer survival in a hospital-based case-only study.

**Materials and methods:**

The relationship between the *MnSOD -102 *C>T polymorphism and survival was examined in a cohort of 291 women who received chemotherapy and/or radiotherapy for incident breast cancer. The *MnSOD -102 *C>T genotype was determined using a TaqMan allele discrimination assay. Patient survival was evaluated according to the *MnSOD *genotype using Kaplan–Meier survival functions. Hazard ratios were calculated from adjusted Cox proportional hazards modeling. All statistical tests were two-sided.

**Results:**

In an evaluation of all women, there was a borderline significant reduction in recurrence-free survival with either one or both variant alleles (CT + TT) when compared with patients with wild-type alleles (CC) (odds ratio, 0.65; 95% confidence interval, 0.42–1.01). When the analysis was restricted to patients receiving radiation therapy, there was a significant reduction in relapse-free survival in women who were heterozygous for the *MnSOD -102 *genotype (relative risk, 0.40; 95% confidence interval, 0.18–0.86). Similarly, when the homozygous and heterozygous variant genotypes were combined, there remained a significant reduction in relapse-free survival in this group (hazard ratio, 0.42; 95% confidence interval, 0.20–0.87).

**Conclusion:**

The *MnSOD -102 *variant allele appears to be associated with an improved recurrence-free survival in all patients, and more dramatically in subjects who received adjuvant radiation therapy.

## Introduction

Breast cancer represents one of the most common cancers among women residing in the United States and worldwide [1,2]. In 2005, 145,920 US residents will be diagnosed with breast cancer [3]. The etiological risk factors common to breast cancer include age, personal or family history, obesity, and lifetime exposure to exogenous and endogenous estrogens.

Genetic risk factors have also been identified, such as the high-penetrance cancer susceptibility genes, *BRCA1 *and *BRCA2*, but these genes only account for 5% of all breast cancer cases [4,5]. It has therefore been established that relatively common genes acting together with endogenous/lifestyle risk factors (low-penetrance genes) are likely to account for a much higher portion of the breast cancer cases together with as yet unidentified high-penetrance genes [6].

The development of breast cancer has been linked to the degree of oxidative stress, particularly with perturbations in the delicate balance between reactive oxygen species (ROS) and oxidative defenses [7]. ROS are generated through the metabolism of estradiol, polyunsaturated fats, ethanol and calories, all which have been considered potential risk factors for breast cancer [8]. Antioxidant enzymes such as superoxide dismutase have been demonstrated to protect cells from oxidative stress. Generation of ROS has been implicated in the etiology of a diversity of human diseases, including cancer [9]. Oxidative stress has been demonstrated to induce cell death as a result of excessive cellular damage associated with lipid peroxidation and alterations of nucleic acids and proteins, triggering apoptosis through the mitochondria [10].

Superoxide dismutase catalyzes the dismutation of the superoxide radical (O_2_^-^) to hydrogen peroxide (H_2_O_2_) and oxygen (O_2_). Three distinct types of superoxide dismutases have been identified in human cells: a homodimeric cytosolic CuZnSOD [11], an extracellular homotetrameric glycosylated superoxide dismutase [12], and a mitochondrial matrix homotetrameric manganese superoxide dismutase (MnSOD) [13].

A specific region of the MnSOD protein is essential for the correct transport and processing of MnSOD by mitochondria. There have been conflicting reports of the association of the polymorphism within the MnSOD mitochondrial targeting sequence (-9 T>C; Val to Ala) and the risk of cancer. A significant gene dose-response relationship has been observed in breast cancer [14,15]; however, more recent reports did not find an association between -9 T>C MnSODand breast cancer [16-18].

Since radiation therapy remains an integral part of the multimodality therapy in the treatment of breast cancer, determining maximal efficacy among patients remains of the utmost importance. Radiation therapy exerts its antitumor effects through the increased formation of ROS, including hydroxyl radicals (OH), hydrogen peroxide (H_2_O_2_) and superoxide anions (O_2_^-^), and its efficacy may be related to the ability to neutralize these effects. Because mitochondrial MnSOD is responsible for catalyzing the conversion of superoxide radicals, there has been a growing belief that gene variants could impact the efficacy of radiation therapy for breast cancer. Similarly, the chemotherapeutic agents most commonly utilized in the treatment of breast cancer, adriamycin and cyclophosphamide, generate ROS that may be effected by MnSOD activity. We recently found that the combination of the *MnSOD -9 *genotype and the myeloperoxidase genotype led to a threefold decrease in the hazard of death among women treated for breast cancer [19]. This association has further strengthened the need to evaluate other possible gene variants that may play a role in therapeutic efficacy.

The polymorphism in the signal sequence, a mutation in the *MnSOD *promoter sequence (*MnSOD -102 *C>T) has been shown to change the binding pattern of AP-2, leading to a reduction in transcriptional activity [20]. We recently reported that this polymorphism is relatively frequent in human populations [21,22]. A relationship between this polymorphism in the *MnSOD *promoter and breast cancer treatment outcomes has not, however, been reported. Similarly, there have been no published reports of the effects the *MnSOD -102 *C>T polymorphism on protein function.

The aim of our study was therefore to investigate the possible associations of the *MnSOD -102 *C>T polymorphism within a case-only study of breast cancer patients to evaluate possible associations with overall and relapse-free survival in relation to the patients' adjuvant therapy.

## Methods

### Study subjects

As previously described [19,23], patients who received their first course of adjuvant treatment for primary invasive breast cancer at the Arkansas Cancer Research Center, University of Arkansas for Medical Sciences from 1985 to 1996 were identified from hospital tumor registry records. Patients with a prior history of cancer were excluded. Hospital tumor registry records were used to obtain information concerning age, stage at diagnosis, tumor size, tumor grade, hormone receptor status, race, and date and type of therapy received (chemotherapy, radiotherapy, surgery, and hormonal treatment). The hospital tumor registry was also the source of follow-up information; the registry conducts active follow-up for each patient, contacting the physician or the patient annually and recording the date last contacted and the vital status. The study protocol was approved by the Institutional Review Board of University of Arkansas for Medical Sciences.

Archived normal lymph nodes or skin, stored in paraffin blocks in the Pathology Department, were used as a source of DNA for genotyping as described previously [19,23]. Hospital tumor registry records identified 815 patients with invasive breast cancer who had been treated at University of Arkansas for Medical Sciences from 1985 to 1996. One hundred and twenty-four (15%) of these patients had received no adjuvant therapy (chemotherapy, radiation therapy, or tamoxifen)and were excluded from the study, as were five (1%) patients with missing adjuvant therapy information. Most of the excluded patients who did not receive adjuvant therapy had lymph node-negative disease.

### DNA extraction and genotyping

Sections (50 μm thick) were cut from archived paraffin normal tissue blocks, the tissue was deparaffinized, and DNA was extracted using a commercially available kit (Qiagen Inc., Valencia, CA, US), as previously described [19,23].

The *MnSOD -102 C>T *polymorphism was determined using a technique developed in our laboratory [21]. Single nucleotide polymorphism-specific PCR primers and fluorogenic probes were designed using Primer Express (version 1.5; Applied Biosystems, Foster City, CA, USA). The fluorogenic probes were labeled with a reporter dye (either FAM or VIC) and are specific for one of the two possible bases (-102 C or -102 T) in the *MnSOD *promoter region. TaqMan Universal PCR Master Mix (Applied Biosystems) was used to prepare the PCR. The two-times mix was optimized for TaqMan reactions and contained AmpliTaq-Gold DNA polymerase, AmpErase, deoxyribonucleotide triphosphate with uracil triphosphate, and a passive reference. Primers, probes, and genomic DNA were added to final concentrations of 300 nM, 100 nM, and 0.5–2.5 ng/μl, respectively.

Controls (no DNA template) were run to ensure there was no amplification of contaminating DNA. The amplification reactions were carried out in an ABI Prism 7700 Sequence Detection System (Applied Biosystems) with two initial hold steps (50°C for 2 min, followed by 95°C for 10 min) and 50 cycles of a two-step PCR (95°C for 15 s, 60°C for 1 min).

The fluorescence intensity of each sample was measured at each temperature change to monitor amplification of the 278-base-pair *MnSOD *promoter region. The -102 nucleotide was determined by the fluorescence ratio of the two single nucleotide polymorphism-specific fluorogenic probes. The fluorescence signal increases when the probe with the exact sequence match binds to the single-stranded template DNA and is digested by the 5'-3' exonuclease activity of AmpliTaq-Gold DNA polymerase (Applied Biosystems). Digestion of the probe releases the fluorescent reporter dye (either FAM or VIC) from the quencher dye.

### Statistical analysis

Crude associations between genotypes for *MnSOD *and overall survival were evaluated using the Kaplan–Meier survival function. The survival time was calculated as the time from diagnosis to death or to the last contact date for living subjects. Both heterozygotes and homozygotes were assessed separately in relation to the referent. Cox proportional hazard models were constructed to assess potential confounding effects of other breast cancer prognostic factors, including age, stage with nodal status, and estrogen and progesterone receptor status. The final multivariate-adjusted models shown include those factors that either changed the estimated effect by 10% or more in a best-fitting model, which was developed by starting with a full model and then excluding covariates that did not improve the overall fit. Survival analysis according to *MnSOD *genotype was conducted separately by treatment group (i.e. chemotherapy or radiation therapy). All analyses were conducted using SAS software (version 8.2; SAS Institute, Inc., Cary, NC, USA). All statistical tests were two sided.

## Results

Acceptable tissue samples and complete baseline information were obtained for 291 cases (Table 1). A slight majority of patients were older than 50 years of age (53%), with a small percentage being of African-American decent (18%). There was an even distribution of patients who were stage 1 (28%), patients who were stage 2 lymph node negative (20%), patients who were stage 2 lymph node positive (30%), patients who were stage 3 (16%), and a smaller percentage who were stage 4 (6%). Fifty-nine percent of estrogen receptor-positive patients received tamoxifen therapy. A large percentage of patients received chemotherapy alone (33%), when compared with those who received radiation alone (9%), with those who received chemotherapy/radiation therapy (19%), and with those who received other combinations (Table 1).

**Figure 1:**
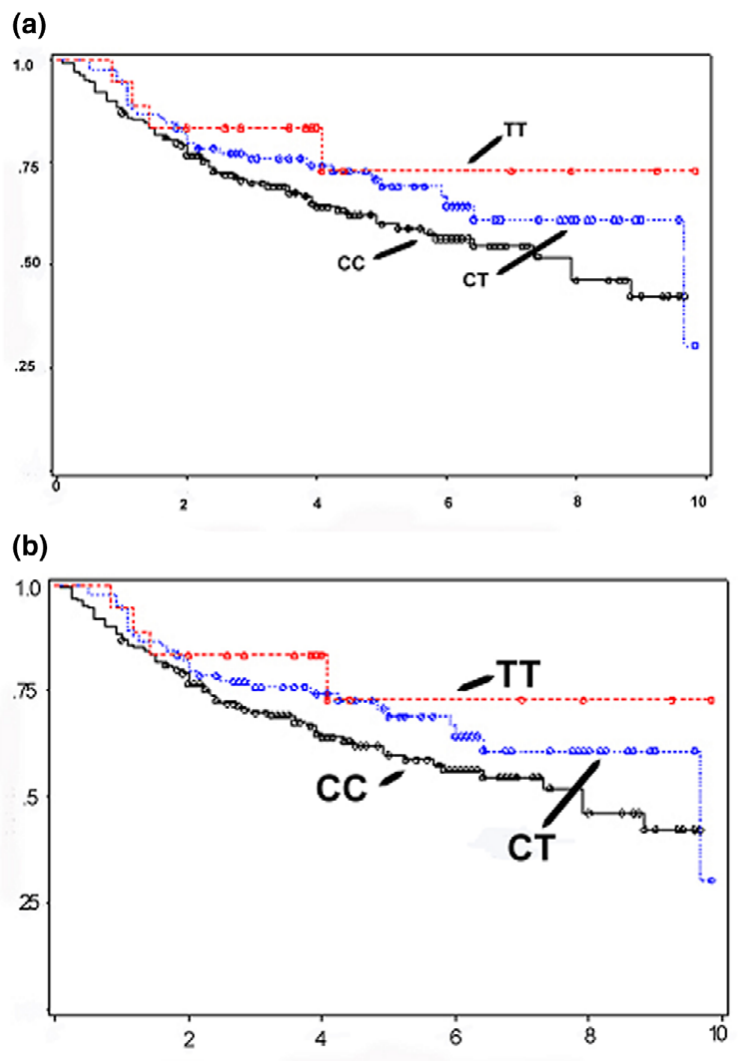
**(a) **Percentage overall survival and **(b) **percentage recurrence-free survival by years of follow-up in the manganese superoxide dismutase -102 genotype. CC, homozygous wild-type genotype; CT, heterozygous for variant genotype; TT, homozygous variant genotype.

In patients with one or two copies of the variant *MnSOD -102 *(CT or TT), there was a nonsignificant impact of genotype on overall survival (Table 2). Similarly, combining patients with one or both variant alleles did not result in a significant change in overall survival when compared with the reference genotype (CC) (Figure 1a). In an evaluation of recurrence-free survival, there was a trend towards significant improvement in recurrence-free survival in patients with one or two variant alleles when compared with women homozygous for the common allele (CC) (odds ratio, 0.65; 95% confidence interval, 0.42–1.01) (Table 2). There was a trend towards an improvement in recurrence-free survival with both homozygous (TT) and heterozygous (CT) variant genotypes (Figure 1b).

Of the study population, there were 160 women who received radiation therapy. Twenty-five patients received radiation alone, 54 patients received radiation and chemotherapy, 46 patients received radiation and tamoxifen, and 35 patients received radiation, chemotherapy, and tamoxifen.

**Figure 2:**
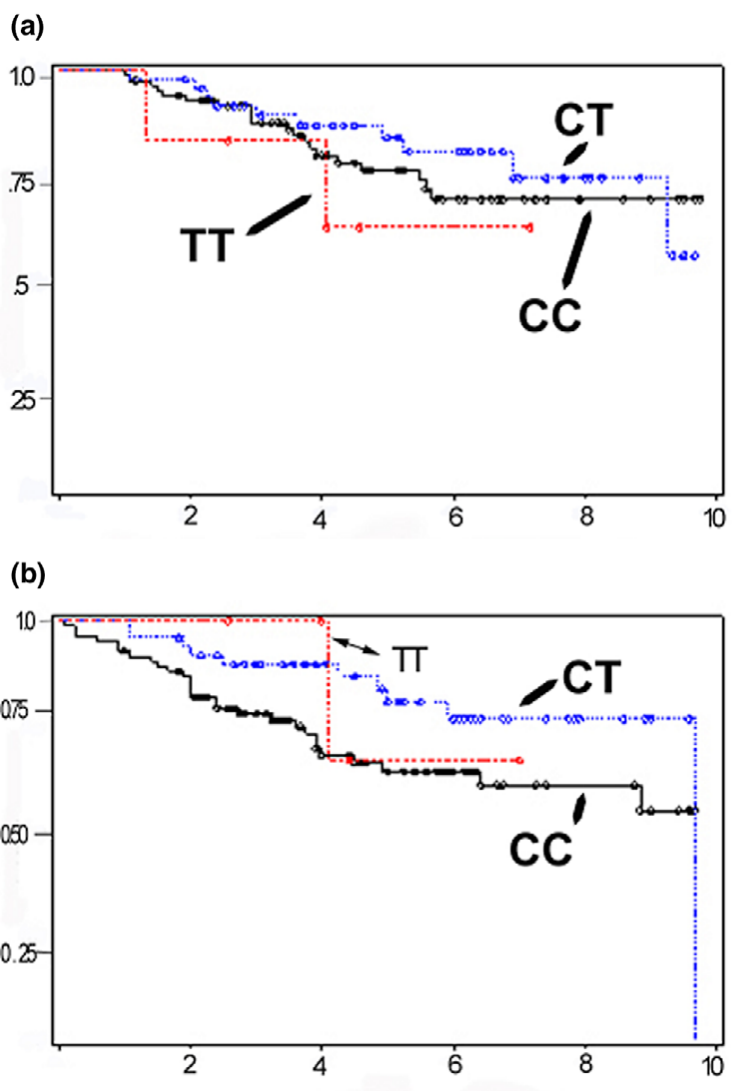
The manganese superoxide dismutase -102 genotype: **(a) **percentage overall survival and **(b) **percentage recurrence-free survival by years of follow-up with radiation therapy. CC, homozygous wild-type genotype; CT, heterozygous for variant genotype; TT, homozygous variant genotype.

In an evaluation of relapse-free survival in subjects receiving radiation therapy there was a significant reduction in relapse-free survival among women who were heterozygous for the *MnSOD -102 *genotype (relative risk, 0.40; 95% confidence interval, 0.18–0.86) (Table 3). When the homozygous and heterozygous variant alleles were combined, there remained a significant reduction in relapse-free survival in this group (Figure 2).

There were 230 patients in the study population who received chemotherapy. Among these women, there was an association of borderline significance between *MnSOD *genotypes and relapse-free survival (adjusted hazard ratio for CT + TT genotype, 0.65; 95% confidence interval, 0.40–1.05) (Table 4). There were, however, no significant associations with overall survival.

## Discussion

In this evaluation of the effects of the *MnSOD -102 *polymorphism on survival after treatment for breast cancer, we found that women with genotypes that would result in less MnSOD expression, and thus higher levels of ROS, had a significant decrease in relapse-free survival among breast cancer subjects who received adjuvant radiation therapy as well as chemotherapy. There was also a trend toward decreased overall relapse-free survival in all subjects with breast cancer. This study further indicates that genetic variants that may result in decreased function, and thus increased oxidative stress, may enhance the efficacy of radiation therapy and thus translate into better disease control. This is the first study to examine the relationship between the *MnSOD -102 *genotype and the survival of breast cancer patients treated with adjuvant therapy.

These results support the our previous findings that demonstrated a decrease in the hazard of death with the *MnSOD -9 *CC and *MPO *GG genotypes [19]. In that analysis, genotypes associated with higher levels of ROS for women with breast cancer treated with adjuvant therapy led to improved overall survival when compared with women with lower levels of ROS.

Our results also add to the already large volume of literature on the role of the mitochondrial MnSOD in cancer cell survival. Evaluation of MnSOD suggests that it is critically important in maintenance of mitochondrial function. Mice with deficiency of this enzyme exhibit progressive cardiomyopathy, neurodegeneration, and perinatal death [24]. These studies also confirmed that transgenic mice that express human MnSOD in the mitochondria are protected from environmental oxygen-induced lung injury [25] and adriamycin-induced cardiac toxicity [26]. In contrast, disruption of the other two superoxide dismutases yielded viable mice that were normal in nonstressful conditions [27]. The mitochondrial MnSOD therefore represents a major cellular defense against oxidative stress.

Additional clinical studies have evaluated MnSOD expression with conflicting results. Janssen and colleagues [28] evaluated 81 gastric adenocarcinomas and found a significantly higher MnSOD expression within the tumor when compared with the normal mucosa. This higher expression did not correlate to a worse outcome; however, the ratio of MnSOD expression of tumor compared with the normal tissue was correlated with a worse overall survival. The significance of this upregulation and worse outcome was felt to be related to chemoresistance but was not elucidated in this study.

A study by Malafa and colleagues [29] reported the difference in MnSOD expression in gastric carcinoma for metastatic and nonmetastatic cancers. Although the nonmetastatic gastric cancers showed no increase in expression versus normal gastric epithelial cells, 93% of primary tumor cells of metastatic gastric cancer cells showed an upregulation of MnSOD enzymatic activity. This correlation of increased MnSOD expression and increased metastatic prevalence correlates with the theory that MnSOD's role changes during the transformation of a cell from normal to dysplasia to cancer. MnSOD initially appears to acts as a tumor suppressor by inhibiting the ROS and preventing cellular damage; however, once the transformation to malignant cells has taken place, the results of several studies infer that the MnSOD acts to protect the malignant cell both from chemotherapy [28,30] and from radiation therapy [31,32] to allow for its progression, replication, and metastasis. This can be explained by the fact that the defenses against malignant cells, whether the body's host defenses or exogenous treatments such as radiation or chemotherapy, often use ROS as a mechanism for cellular destruction. Malignant cells that have an increased expression of MnSOD would therefore be more resistant to cellular destruction and would therefore more probably be resistant to treatment. This increase in MnSOD expression would lead to an increase in the probability of proliferation and metastasis.

The theory has been strengthened by Izutani and colleagues' [31] demonstration of the role of MnSOD after progression to cancer. An upregulation of MnSOD mRNA in gastric carcinoma was demonstrated, which provided a protective mechanism from the cell toxicity of tumor necrosis factor alpha from ROS. Izutani and colleagues' [32] later report confirmed these findings by demonstrating an upregulation of MnSOD mRNA in squamous esophageal carcinoma. This upregulation of MnSOD mRNA in active malignant cells not only acts to protect from the body's host defensive mechanisms, but a recent report by Hur and colleagues [30] demonstrated that MnSOD upregulation also gives gastric cancers protection against current chemotherapy agents. Further research by Izutani and colleagues [33] indicated that MnSOD expression inhibited the tumor sensitivity of adriamycin in esophageal and gastric cancers. Furthermore, use of transforming growth factor beta to inhibit MnSOD showed an increase in the effectiveness of adriamycin as a suppressor of these tumors [33]. Not only does this research solidify the theory that MnSOD protects the tumor cells after their transformation, but it leads us to believe that, by inhibiting MnSOD, chemotherapy may be made more effective.

Our results are in contrast to a smaller study (*n *= 80) of women receiving radiation therapy for breast cancer, who found no effects of the *MnSOD -9 *polymorphisms on clinically detectable skin reactions to therapy [34]. These discrepant results may be due to more dominant effects that the *MnSOD -102 *polymorphism plays in MnSOD function, or could be due to differential effects of MnSOD variability on normal and tumor tissue or, finally, could be due to the small number of subjects (*n *= 80) in the study. Since the *MnSOD -9 *polymorphism leads to partial inner mitochondrial membrane transport arrest [35], there have been conflicting reports as to which polymorphism (c allele = alanine or t allele = valine) leads to the greater risk of cancer. Since the *MnSOD -102 *polymorphism affects the AP-2 binding and thus reduces transcriptional activity, however, there may be a greater effect from this polymorphism than from other polymorphisms that have been reported.

Even with this report indicating the efficacy effects of ROS producing therapies and the relationship to *MnSOD *polymorphisms, this initial evaluation needs to be replicated in a larger study, with greater homogeneity of the subject population. Since very little is know about the clinical effects of the *MnSOD -102 *polymorphism in carcinogenesis, tumor cell resistance and chemotherapy/radiation therapy efficacy, further evaluation is needed. Because this study involved women who received various adjuvant treatments (radiation, chemotherapy and/or hormonal therapy), we are unable to establish the effects that each individual therapy had on overall survival and on relapse-free survival. Since there is a similarity in the effects that chemotherapy in breast cancer treatment and radiation therapy induce (generation of ROS), however, it is less likely that individual treatment evaluation will lead to different results. Radiation therapy and the chemotherapeutic agents most commonly utilized in the treatment of breast cancer, adriamycin and cyclophosphamide, exert their antitumor effects through the increased formation of ROS, including hydroxyl radicals (OH), hydrogen peroxide (H_2_O_2_), and superoxide anions (O_2_^-^), and the efficacy may be related to the ability of the patients'/tumors' ability to neutralize these effects. Since MnSOD is not involved in the metabolism of any specific chemotherapeutic agent we believe these results will remain consistent, but they need to be evaluated in a larger study.

## Conclusion

In summary, this reports demonstrates the first evaluation that decreases in MnSOD by the variant genotype are associated with improved relapse-free survival in women treated for breast cancer.

## Abbreviations

MnSOD = manganese superoxide dismutase; PCR = polymerase chain reaction; ROS = reactive oxygen species.

## Competing interests

The authors declare that they have no competing interests.

## Authors' contributions

RM participated in design of the study and manuscript preparation. JA participated in the statistical analysis. SN contributed to the study design and to the analysis and interpretation of the data. DH participated in design of the study and manuscript preparation. MD and BM participated in the design of the assay methods. CA contributed to the study design, analysis, and interpretation of the data, and to manuscript preparation.

## Figures and Tables

**Table 1:** Demographic information and pathological characteristics of study participants and distribution of characteristics

	*n *(%)
Age at diagnosis	
≤50 years	133 (46)
>50 years	158 (54)
Race	
Caucasian	239 (82)
African-American	52 (18)
Stage and node status	
Stage 1	82 (28)
Stage 2 negative	57 (20)
Stage 2 positive	87 (30)
Stage 3	48 (16)
Stage 4	17 (6)
Estrogen receptor status	
Positive	176 (64)
Negative	101 (36)
Progesterone receptor status	
Positive	128 (46)
Negative	148 (54)
Treatment	
Chemotherapy only	95 (33)
Radiation only	25 (9)
Chemotherapy/radiation	54 (19)
radiation/tamoxifen	36 (12)
Chemotherapy/tamoxifen	44 (16)
Chemotherapy/radiation/tamoxifen	35 (12)

Total *n *= 291.

**Table 2:** Manganese superoxide dismutase -102 genotype: overall and recurrence-free survivals

Genotype	Cases	Deaths	Hazard ratio minimally adjusted^a ^(95% confidence interval)	Hazard ratio fully adjusted^b ^(95% confidence interval)
Overall survival	291	85	*P *for trend = 0.62	*P *for trend = 0.88
CC	180	55	1 (reference)	1 (reference)
CT	92	25	0.89 (0.55–1.43)	0.75 (0.465–1.24)
TT	19	5	0.88 (0.35–2.20)	1.06 (0.42–2.69)
				
CC	180	55	1 (reference)	1 (reference)
CT + TT	111	30	0.89 (0.57–1.38)	0.80 (0.50–1.28)
Recurrence-free survival	280^c^	100	*P *for trend = 0.12	*P *for trend = 0.08
CC	175	68	1 (reference)	1 (reference)
CT	87	28	0.77 (0.50–1.20)	0.65 (0.41–1.04)
TT	18	4	0.55 (0.20–1.50)	0.66 (0.24–1.83)
				
CC	175	68	1 (reference)	1 (reference)
CT + TT	105	32	0.73 (0.48–1.12)	0.65 (0.42–1.01)

CC, homozygous for the wild-type manganese superoxide dismutase gene; CT, heterozygous for the variant genotype; TT, homozygous for the variant genotype. ^a^Minimally adjusted model: age. ^b^Fully adjusted model: age, stage with node status at diagnosis, race, estrogen receptor status, and progesterone receptor status. ^c^Eleven subjects were never disease free, thus they were deleted for recurrence-free survival.

**Table 3:** Manganese superoxide dismutase -102 genotype: overall and recurrence-free survivals with radiation therapy

Genotype	Cases	Deaths	Hazard ratio minimally adjusted^a ^(95% confidence interval)	Hazard ratio fully adjusted^b ^(95% confidence interval)
Overall survival	150	33	*P *for trend = 0.67	*P *for trend = 0.91
CC	93	20	1 (reference)	1 (reference)
CT	51	11	0.97 (0.46–2.04)	0.74 (0.35–1.58)
TT	6	2	1.95 (0.45–8.49)	2.23 (0.46–10.79)
				
CC	93	20	1 (reference)	1 (reference)
CT + TT	57	13	1.05 (0.52–2.12)	0.82 (0.40–1.70)
Recurrence-free survival	145	40	*P *for trend = 0.14	*P *for trend = 0.03
CC	90	29	1 (reference)	1 (reference)
CT	50	10	0.57 (0.28–1.18)	0.40 (0.18–0.86)
TT	5	1	0.60 (0.08–4.47)	0.69 (0.09–5.46)
				
CC	90	29	1 (reference)	1 (reference)
CT + TT	55	11	0.57 (0.28–1.16)	0.42 (0.20–0.87)

CC, homozygous for the wild-type manganese superoxide dismutase gene; CT, heterozygous for the variant genotype; TT, homozygous for the variant genotype. ^a^Minimally adjusted model: age. ^b^Fully adjusted model: age, stage with node status at diagnosis, race, estrogen receptor status, and progesterone receptor status.

**Table 4:** Manganese superoxide dismutase -102 genotype: overall and recurrence-free survivals with adjuvant chemotherapy

Genotype	Cases	Deaths	Hazard ratio minimally adjusted^a ^(95% confidence interval)	Hazard ratio fully adjusted^b ^(95% confidence interval)
Overall survival	230	75	*P *for trend = 0.46	*P *for trend = 0.42
CC	140	49	1 (reference)	1 (reference)
CT	73	21	0.83 (0.49–1.38)	0.67 (0.38–1.17)
TT	17	5	0.81 (0.32–2.04)	1.07 (0.42–2.74)
				
CC	140	49	1 (reference)	1 (reference)
CT + TT	90	26	0.82 (0.51–1.33)	0.73 (0.44–1.22)
Recurrence-free survival	220	85	*P *for trend = 0.16	*P *for trend = 0.12
CC	136	57	1 (reference)	1 (reference)
CT	68	24	0.79 (0.49–1.28)	0.64 (0.38–1.07)
TT	16	4	0.55 (0.20–1.52)	0.73 (0.26–2.049)
				
CC	136	57	1 (reference)	1 (reference)
CT + TT	84	28	0.75 (0.47–1.18)	0.65 (0.40–1.05)

CC, homozygous for the wild-type manganese superoxide dismutase gene; CT, heterozygous for the variant genotype; TT, homozygous for the variant genotype. ^a^Minimally adjusted model: age. ^b^Fully adjusted model: age, stage with node status at diagnosis, race, estrogen receptor status, and progesterone receptor status.
